# Research on Dual-Frequency Electromagnetic False Alarm Interference Effect of a Typical Radar

**DOI:** 10.3390/s22093574

**Published:** 2022-05-07

**Authors:** Xue Du, Guanghui Wei, Kai Zhao, Hongze Zhao, Xuxu Lyu

**Affiliations:** 1 National Key Laboratory on Electromagnetic Environmental Effects, Shijiazhuang Campus of Army Engineering University, Shijiazhuang 050003, China; wei-guanghui@sohu.com (G.W.); z_hongze@126.com (H.Z.); lyuxuxu@outlook.com (X.L.); 2 63870 Troops, Huayin 714200, China; oeczhao@126.com

**Keywords:** radar false alarm signal, step frequency radar, continuous wave electromagnetic interference, false alarm signal position

## Abstract

In order to master the position variation rule of radar false alarm signal under continuous wave (CW) electromagnetic interference and reveal the mechanism of CW on radar, taking a certain type of stepping frequency radar as the research object, theoretical analysis of the imaging mechanism of radar CW electromagnetic interference false alarm signals from the perspective of time-frequency decoupling and receiver signal processing. Secondly, electromagnetic interference injection method is used to test the single-frequency and dual-frequency electromagnetic interference effect of the tested equipment. The results show that under the single frequency CW electromagnetic interference, the sensitive bandwidth of false alarm signal is about ±75 MHz, and the position of false alarm signal irregularity changes. Under the in-band dual-frequency CW electromagnetic interference, the position of non-intermodulation false alarm signal is similar to that of single frequency. However, the distance difference of two non-intermodulation false alarm signals is regular. In addition, the positions of the second-order intermodulation false alarm signals of the tested radar are also regular, and its position changes with the change of the second-order intermodulation frequency difference.

## 1. Introduction

Radar uses electromagnetic waves to detect targets, which play a very important role in the informationized battlefield. They are widely used in unmanned combat systems with its advantages of all-weather and real-time acquisition of target information [1,2]. With the widespread use of high-tech electromagnetic equipment, the electromagnetic environment is becoming increasingly complex, which seriously affects the normal effectiveness of the equipment [3,4]. In order to improve the anti-electromagnetic interference ability of radar equipment, the law of electromagnetic interference blocking effect of radar equipment is studied in depth in the early stage, and the law and mechanism of blocking interference effect inside and outside the radar band are determined [3,5]. The prediction model of multi-frequency non-intermodulation electromagnetic interference blocking effect in complex electromagnetic environment is established [6,7]. However, continuous wave electromagnetic interference will cause false alarm effects, in addition to blocking the effect of radar [8]. For example, reference [9,10,11,12] used digital reconstruction technology to analyze the causes of false target signals and proposed corresponding anti-interference methods. Reference [13] analyzed the false signals formed by the storage and forwarding of received radar signals by the system. Reference [14] studied the influence of noise on false signals. Reference [2] proposed modeling and evaluation of false signals based on visual consistency. At present, the research on radar false alarm effect is mostly based on information jamming, and the research on non-information jamming is less than information jamming. Reference [8] pointed out that single-frequency CW electromagnetic interference would cause blocking interference and false alarm interference to the radar, and the paper analyzed that the false alarm signal level under radar single-frequency electromagnetic interference showed a trend of “increasing first and then slowing down” with the increase of interference field strength but did not analyze its target location. The randomness of the false alarm signal position makes the radar greatly affected in detecting the target distance. Therefore, it is of great military significance to study the effect law of false alarm signals in complex electromagnetic environment to evaluate the adaptability of radar equipment in complex electromagnetic environments.

## 2. Imaging Mechanism of False Alarm Signal

### 2.1. Time-Frequency Decoupling Angle Analysis

The radar transmit signal is expressed as:

(1) $$u\left(t\right)=\sum_{k=1}^{N-1}rect(\frac{t}{T_{r}})e^{-j\left[2\pi \left(f_{l}+k\Delta f\right)t+\theta_{k}\right]}$$

where *T_r_* is the sub-period of transmitting pulse, *θ_k_* is the initial phase of signal, is the initial frequency of signal, Δ*f* is the step interval of frequency, and *N* is the number of sub-periods. The transmitting signal detects the target backscatter and enters the receiver, and the echo signal of the target is received in the Kth sub-period.

(2) $$u_{r}\left(t,k\right)=u_{k}\left(t-\tau_{k}\right)=\alpha (k)rect(\frac{t-\tau_{k}}{T_{r}})e^{-j\left[2\pi \left(f_{l}+k\Delta f\right)(t-\tau_{k})+\theta_{k}\right]}$$

where *τ_k_* is the target echo delay; for the stationary target $\tau_{k}=2R/c$, *R* is the detection target distance, α(k) is the amplitude of the received target signal in the *K*th sub-period. Assuming that the *k* + 1 sub-period is subjected to the interference signal with similar frequency, the interference signal and the target echo signal are mixed together, and the filter cannot effectively filter the interference signal, at this time the interference signal and the target echo signal are mixed together, the filter cannot be effectively filtered out the interference signal, the received signal contains the target signal and interference signal, which is expressed as

(3) $$u_{r}\left(t,k+1\right)=u_{k+1}\left(t\right)+u_{{}_{k+1}}^{\prime }\left(t\right)+n_{k+1}(t)$$

where n(t) is gaussian noise, $u_{k}^{\prime }\left(t\right)\gg n(t)$, and in order to simplify the derivation process, the n(t) component is temporarily ignored. Assume that the single frequency electromagnetic interference signal

(4) $$u_{k}^{\prime }\left(t\right)=rect(\frac{t}{T_{r}})e^{-j2\pi \left(f_{j}t+\varphi_{j}\right)}=e^{-j\left[2\pi (f_{l}+f_{d})t+\varphi_{jk}\right]}$$

where *f_d_* is the frequency component of the interference signal relative to the initial signal frequency, and *φ_jk_* is the initial phase of the interference signal. Received interference signals are expressed as

(5) $$u_{k}^{\prime }\left(t-\tau_{k}^{\prime }\right)=\beta (k)rect(\frac{t-\tau_{k}^{\prime }}{T_{r}})e^{-j\left[2\pi (f_{l}+f_{d})(t-\tau_{k}^{\prime })+\varphi_{jk}\right]}$$

where, $\tau_{k}^{\prime }$ is the delay of interference signal to receiver, β(k) is the amplitude of interference signal received in the *K*th sub-period, and the signal received in the *k* + 1 sub-period is expressed as

(6) $$u_{r}\left(t,k+1\right)=u_{k+1}\left(t-\tau_{k+1}\right)+u_{k+1}^{\prime }\left(t-\tau_{k+1}^{\prime }\right)$$

where $\tau_{k+1},\tau_{k+1}^{\prime }$ represent target echo signal and interference signal target delay of the *k* + 1 sub-pulse, respectively. The false target signal generated by electromagnetic interference is defined as a false alarm signal. Time-frequency decoupling is performed for the received signal (6), that is, using $u_{r}\left(t,k\right)$ to Stretch $u_{r}\left(t,k+1\right)$ is expressed as

(7) $$u_{r}^{*}\left(t,k+1\right)u_{r}\left(t,k\right)=u_{k}\left(t-\tau_{k}\right)u_{k+1}^{*}\left(t-\tau_{k+1}\right)+u_{k}\left(t-\tau_{k}\right)u_{k+1}^{\prime *}\left(t-\tau_{k+1}^{\prime }\right)$$

Assuming sampling time $t_{k}=kT_{r}$, that is $k=Floor\left(\frac{t_{k}}{T_{r}}\right)$, which is obtained by combining (3), (4), (6) and (7)

(8) $$\begin{array}{ll} u_{r}^{*}\left(t,k+1\right)u_{r}\left(t,k\right) & =\alpha (k)\alpha (k+1)rect(\frac{t_{k}-\tau_{k}}{T_{r}})rect(\frac{t_{k}-\tau_{k+1}}{T_{r}})e^{-j\varphi_{k+1}}\\ & +\alpha (k)\beta (k+1)rect(\frac{t_{k}-\tau_{k}}{T_{r}})rect(\frac{t_{k}-\tau_{k+1}^{\prime }}{T_{r}})e^{-j\varphi_{{}_{k+1}}^{\prime }} \end{array}$$

In Formula (8), $\varphi_{k+1},\varphi_{k+1}^{\prime }$ are represented as follows

(9) $$\{\begin{array}{c} \varphi_{k+1}=2\pi k\Delta f\left(\tau_{k+1}-\tau_{k}-T_{r}\right)+f_{l}\left(\tau_{k+1}-\tau_{k}\right)+\Delta f\tau_{k+1}-\left(\theta_{k+1}-\theta_{k}\right)\\ \varphi_{k+1}^{\prime }=2\pi \left[k\Delta ft_{k}-k\Delta f\tau_{k}-f_{dk}t_{k}-f_{l}t_{k}-f_{d(k+1)}t_{k}+\left(\tau_{k+1}^{\prime }-\tau_{k}\right)f_{l}+f_{d(k+1)}\tau_{k+1}^{\prime }\right]+\left(\theta_{k}-\varphi_{j\left(k+1\right)}\right) \end{array}$$

Equation (8) is simplified, and the result is expressed as

(10) $$\begin{array}{ll} u_{r}^{*}\left(t,k+1\right)u_{r}\left(t,k\right) & =\alpha (k)\alpha (k+1)rect(\frac{t_{k}-\tau_{k}}{T_{r}})rect(\frac{t_{k}-\tau_{k+1}}{T_{r}})e^{-j\psi_{k+1}}\\ & +\alpha (k)\beta (k+1)rect(\frac{t_{k}-\tau_{k}}{T_{r}})rect(\frac{t_{k}-\tau_{k+1}^{\prime }}{T_{r}})e^{-j\psi_{k+1}^{\prime }} \end{array}$$

In Formula (9), $\varphi_{k+1},\varphi_{k+1}^{\prime }$ are represented as follows

(11) $$\{\begin{array}{c} \psi_{k+1}=2\pi \left[\left(\tau_{k+1}-\tau_{k}-T_{r}\right)\Delta fk+f_{l}\left(\tau_{k+1}-\tau_{k}\right)+\Delta f\tau_{k+1}\right]-\left(\theta_{k+1}-\theta_{k}\right)\\ \psi_{k+1}^{\prime }=2\pi \left[\Delta fT_{r}k^{2}-\Delta f\tau_{k}k+\left(f_{dk}+f_{l}+f_{d(k+1)}\right)kT_{r}+f_{l}\left(\tau_{k+1}^{\prime }-\tau_{k}\right)+\left(f_{j}-f_{l}\right)\tau_{k+1}^{\prime }-\varphi_{jk}\right]+\theta_{k} \end{array}$$

Assuming that the amplitude of the signal is a unit value, for the stationary target *R*, it is considered as $\tau_{k+1}\approx \tau_{k+1}^{\prime }\approx \tau_{k},\theta_{k+1}\approx \theta_{k}$ at the moment of very small change, the first item of Equation (9) shows that the detection target signal contains a primary phase of *k*, which can be regarded as a frequency domain signal with a time point of 1 and a linear change of frequency, and subsequently processed by IFFT transform to obtain the target signal distance value. The second term is the false alarm signal generated by electromagnetic interference contains *k*^2^ quadratic phase, which causes the interference signal energy divergence, waveform broadening. The primary phase containing k can be regarded as a frequency domain signal with a linear frequency change at the time node *T_r_*, and a fixed false alarm signal is generated by signal processing. For the out-of-band fixed frequency interference signal $f_{dk}=f_{d\left(k+1\right)}$, $\theta_{k}$ is the initial phase of each sub-period of the transmitted signal, and its different values have different effects on the false alarm target.

### 2.2. Analysis of Signal Processing Angle of Receiver

The imaging mechanism of false alarm signal is explained from the perspective of receiver mixing. Assume that a single frequency CW electromagnetic interference signal

(12) $$u_{k}^{\prime }\left(t\right)=e^{-j2\pi \left(f_{j1}t+\varphi_{j1}\right)}$$

Suppose the local vibration signal

(13) $$u_{L}\left(t\right)=2\sum_{i=0}^{N-1}rect\left(\frac{t}{T_{r}}\right)e^{-j\left[2\pi \left(f_{l}+k\Delta f\right)t+\theta_{k}\right]}$$

When the equipment is disturbed, assuming that the echo signal amplitude is a unit value, the received signal is expressed as

(14) $$\begin{array}{ll} S_{r}\left(k\right)+S_{j}\left(k\right) & =S_{r1}\left(k\right)\cdot S_{r2}\left(k\right)+S_{j1}\left(k\right)\cdot S_{j2}\left(k\right)\cdot S_{j3}\left(k\right)\cdot S_{j4}\left(k\right)\\ & =e^{-j2\pi f_{l}\tau_{k}}\cdot e^{-j2\pi \tau_{k}\Delta fk}+e^{-j2\pi \left(f_{l}T_{r}-f_{j1}T_{r}\right)k}\cdot e^{-j\theta_{k}}\cdot e^{-j2\pi \Delta fT_{r}k^{2}}\cdot e^{{}_{j\varphi_{j1}}} \end{array}$$

In Equation (14), the first term $e^{-j2\pi f_{l}\tau_{k}}$ is a constant, which has no influence on the distance. The second term $e^{-j2\pi \tau_{k}\Delta fk}$ can be regarded as a frequency domain signal with linear frequency change, which will generate the range image at a fixed position, that is, a useful target echo signal. Therefore, the results obtained by IFFT operation on the second term are expressed as follows

(15) $$S_{r2}\left(n\right)=\frac{1}{N}\sum_{k=0}^{N-1}S_{r2}\left(k\right)e^{\frac{j2\pi kn}{N}}$$

Let $l=Round\left(N\Delta f\tau_{k}\right)$ and further calculate Equation (15)

(16) $$\begin{array}{l} S_{r2}\left(n\right)=\frac{1}{N}\sum_{k=0}^{N-1}S_{r2}\left(k\right)e^{\frac{j2\pi kn}{N}}=\frac{1}{N}\sum_{k=0}^{N-1}e^{\frac{j2\pi k\left(n-l\right)}{N}}=\frac{1}{N}\cdot \frac{1-e^{j2\pi k\left(n-l\right)}}{1-e^{\frac{j2\pi k\left(n-l\right)}{N}}}\\ =\frac{1}{N}\cdot \frac{e^{j\pi \left(n-l\right)}}{1-e^{\frac{j\pi \left(n-l\right)}{N}}}\cdot \frac{e^{-j\pi \left(n-l\right)}-e^{j\pi \left(n-l\right)}}{e^{\frac{-j\pi \left(n-l\right)}{N}}-e^{\frac{j\pi \left(n-l\right)}{N}}}\\ =\frac{1}{N}\cdot \frac{\sin\pi \left(n-l\right)}{\sin\left(\frac{\pi \left(n-l\right)}{N}\right)}\cdot e^{\frac{j\pi \left(N-1\right)\left(n-l\right)}{N}}\\ =\frac{\sin c\left(n-l\right)}{\sin c\frac{\left(n-l\right)}{N}}\cdot e^{\frac{j\pi \left(N-1\right)\left(n-l\right)}{N}} \end{array}$$

When n=l, $\left|S_{r2}\left(n\right)\right|$ takes the maximum, the target position is expressed as

(17) $$R_{0}=\frac{cn}{2N\Delta f}$$

In Equation (14), $e^{-j2\pi \tau_{k}\Delta fk}$ makes the distance image at a fixed position, $e^{-j\theta_{k}}$ causes the position of the false alarm signal to move on the basis of a fixed position, $S_{j3}\left(k\right)$ contains a quadratic term of *k*, which widens the signal waveform and diverges the energy, and $S_{j4}\left(k\right)$ has no effect on the distance. The position change of the false alarm signal is mainly related to the primary item and the uncertainty item of *k*. Let $p=Round\left[N\left(f_{l}T_{r}-f_{j1}T_{r}\right)\right]$, the result of IFFT on the primary item of *k* is expressed as

(18) $$\begin{array}{l} S_{j1}\left(n\right)=\frac{1}{N}\sum_{k=0}^{N-1}S_{j1}\left(k\right)e^{\frac{j2\pi kn}{N}}\\ =\frac{1}{N}\cdot \frac{\sin\pi \left(n-p\right)}{\sin\left(\frac{\pi \left(n-p\right)}{N}\right)}\cdot e^{\frac{j\pi \left(N-1\right)\left(p-l\right)}{N}}\\ =\frac{\sin c\left(n-p\right)}{\sin c\frac{\left(n-p\right)}{N}}\cdot e^{\frac{j\pi \left(N-1\right)\left(p-l\right)}{N}} \end{array}$$

Let $q=\theta_{k}$, IFFT operations for variable phase

(19) $$\begin{array}{l} S_{j\theta }\left(n\right)=\frac{1}{N}\sum_{k=0}^{N-1}S_{j2}\left(k\right)e^{\frac{j2\pi kn}{N}}\\ =\frac{1}{N}\cdot e^{-j\theta_{k}}\cdot \sum_{k=0}^{N-1}e^{\frac{j2\pi kn}{N}}\\ =\frac{\sin c\left(n\right)}{\sin c\left(\frac{n}{N}\right)}\cdot e^{-j\theta_{k}}\cdot \frac{e^{j\pi n}}{e^{j\pi \frac{n}{N}}} \end{array}$$

When p+q=l, calculated false alarm signal position

(20) $$R_{j1}=\frac{c\left(f_{l}-f_{j1}\right)T_{r}}{2\Delta f}+\frac{c\theta_{k}}{2N\Delta f}$$

From the above analysis, it can be seen that when *f_j_*_1_ is close to the receiver RF front-end filter of *f_s_* and cannot be effectively filtered, *f_j_*_1_ and *f_s_* are together into the receiver for mixing, amplification, and signal processing. The position of false alarm signal is obtained by combining IFFT operation and Euler equation. IFFT operation can also be performed on (9), and the analysis method is consistent.

Combined with the research conclusion of distance folding in References [15,16], Equation (20) is further derived to obtain the position expression in the real measurement of false alarm target. When the tested radar satisfies the tight constraint condition [16], the unambiguous distance corresponding to the sub-period of the transmitting signal is *r_τ_* = *cT_r_*/2.

(21) $$R_{j1}^{\prime }=R_{j1}-r_{\tau }\mathrm{Floor}\left(\frac{R_{j}}{r_{\tau }}\right)$$

where Floor(x) is the downward integral function.

## 3. Interference Mechanism of Dual Frequency Electromagnetic False Alarm

### 3.1. Imaging Mechanism of Dual Frequency Non-Intermodulation False Alarm Signal

Assuming dual-frequency electromagnetic interference signal

(22) $$u_{kj}^{\prime }\left(t\right)=e^{-j2\pi \left(f_{1}t+\varphi_{j1}\right)}+e^{-j2\pi \left(f_{2}t+\varphi_{j2}\right)}$$

Without considering the intermodulation factor, the dual frequency interference component 2 is sampled by mixing and the signal obtained is

(23) $$e^{-j\left[2\pi \Delta fT_{r}k^{2}-\varphi_{j2}+2\pi \left(f_{l}T_{r}-f_{j2}T_{r}\right)k+\theta_{k}\right]}$$

Assuming that the amplitude is a unit value, combining with Equations (22) and (25) shows the false alarm target formed by interference component 2

(24) $$R_{j2}=\frac{c\left(f_{l}-f_{j2}\right)T_{r}}{2\Delta f}+\frac{c\theta_{k}}{2N\Delta f}$$

The actual location is

(25) $$R_{j2}^{\prime }=R_{j2}-r_{\tau }\mathrm{Floor}\left(\frac{R_{j2}}{r_{\tau }}\right)$$

Combining Equations (25) and (21), dual frequency electromagnetic radiation directly formed by the distance difference between the two false alarm targets is

(26) $$\Delta R=R_{j2}^{\prime }-R_{j1}^{\prime }=\left(R_{j2}-R_{j1}\right)-r_{\tau }\left[\mathrm{Floor}\left(\frac{R_{2}}{r_{\tau }}\right)-\mathrm{Floor}\left(\frac{R_{1}}{r_{\tau }}\right)\right]$$

It can be seen from the above analysis that dual frequency electromagnetic interference will cause the tested radar to produce two non-intermodulation false alarm signals. The position of a single false alarm signal is uncertain, but the distance difference between the two is regular. 

### 3.2. Imaging Mechanism of Second Order Intermodulation False Alarm Signal

When the dual frequency interference frequency is close to the working frequency, due to the effect of nonlinear devices, the two will produce the second-order intermodulation interference component $f_{j2}-f_{j1}$, which can pass through the low-pass filter together with the useful signal, and form the second-order intermodulation false alarm target signal through subsequent signal processing. 

When the interference frequency *f_j_*_1_*, f_j_*_2_ are close to the working frequency, due to the nonlinearity of the mixing circuit, *f_j_*_1_, *f_j_*_2_ in the mixing circuit will generate the second-order intermodulation false alarm signal. Combined with Equation (25), obtain the second-order intermodulation false alarm signal as

(27) $$u_{j2}\left(t\right)=e^{-j\left[2\pi \left(f_{j2}-f_{j1}\right)t+\left(\varphi_{j}{}_{2}-\varphi_{j}{}_{1}\right)\right]}$$

This signal is sampled, and the signal is expressed as

(28) $$u_{j2}\left(t\right)=e^{-j\left[2\pi \left(f_{j2}T_{r}-f_{j1}T_{r}\right)k\right]}\cdot e^{-j\left(\varphi_{j2}-\varphi_{j1}\right)}$$

From Equation (28), the second-order intermodulation false alarm signal does not contain the second phase of *k*, so the waveform shows a “spike” type. Similarly, let $m=\mathrm{Round}\left[N\left(f_{j2}T_{r}-f_{j1}T_{r}\right)\right]$, Equation (28) for the IFFT operation to obtain

(29) $$S_{j2}\left(n\right)=\frac{\sin c\left(n-m\right)}{\sin c\frac{\left(n-m\right)}{N}}\cdot e^{\frac{j\pi \left(N-1\right)\left(n-m\right)}{N}}$$

When *n = m*, the value of Equation (29) takes the maximum value, and m is substituted into Equation (19). The second-order intermodulation signal formed by the false alarm target distance is expressed as

(30) $$R_{jm2}\approx \frac{\left|f_{j1}-f_{j2}\right|cT_{r}}{2\Delta f}$$

From the above analysis, the dual-frequency electromagnetic interference law of the tested radar can be summarized as shown in Table 1.

## 4. Electromagnetic False Alarm Interference Effect Test and Results

### 4.1. Pre-Test Preparation

**Figure 1:**
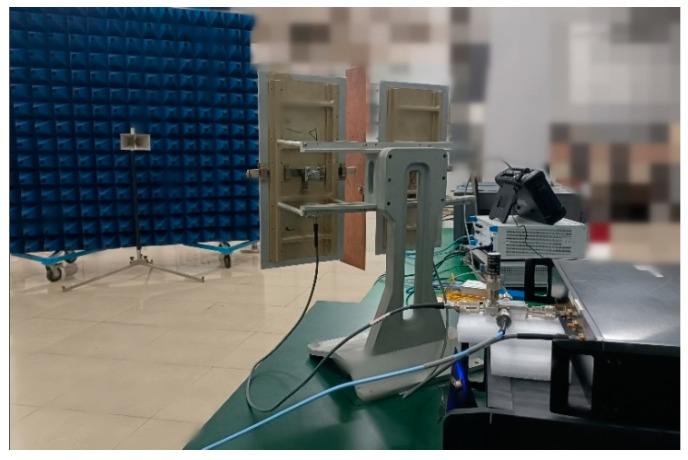
Block diagram of field configuration.

Electromagnetic interference effect test was carried out on the tested radar by electromagnetic injection method. Strictly speaking, electromagnetic injection and irradiation are not completely equivalent, but for the radar test system, electromagnetic interference is mainly coupled to the RF front end by antenna [17,18,19,20,21]. Reference [18] analyzed the radiation equivalent process and test process of the tested equipment in detail, which is not repeated here. The signal source generates the dual-frequency CW interference signal connected to the microwave power amplifier through the combiner, and then the interference signal is injected into the RF front end of the test equipment through the injection module. The spectrometer monitors the input power of the interference signal in real time; the working frequency of the tested radar is *f*_0_, the working bandwidth is *f*_0_ ± 100 MHz, and the maximum display range of the distance window is 5000 m. The field configuration block diagram is shown in Figure 1.

The main parameters of the equipment are as follows: The signal generator uses ROHDE & SCHWARZ SMR 20, which can generate 1~20 GHz microwave signal. The power amplifier uses AR 200 T, working frequency band 7.5~18 GHz, and maximum output power 200 W. The directional coupler matched with the power amplifier, and the coupling degree of the forward power monitoring port is 50 dB. The spectrum analyzer uses Agilent company’s E7405A and Ceyear company’s 4204 G, and the frequency ranges are 100 Hz~26.5 GHz and 9 KHz~44 GHz. The target antenna uses BBHA 9120D type dual ridge broadband horn antenna, a frequency range of 1 to 18 GHz, and a gain of 6.3 to 18 dBi. In addition, the method of using the injection coupling module with monitoring function was developed by the team earlier [17].

### 4.2. Electromagnetic Sensitive Frequency Band Test and Result Analysis of False Alarm Interference

**Figure 2:**
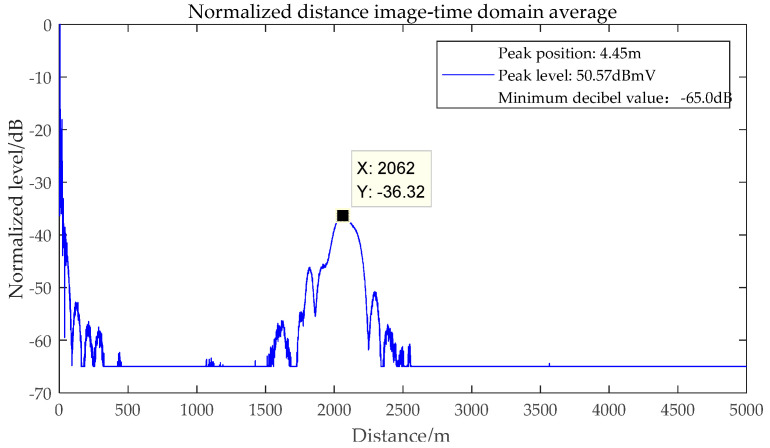
One-dimensional range image of single frequency electromagnetic interference false alarm signal.

Combining GJB8848-2016 and GJB 151B-2013 [22,23], set the frequency offset of the signal source as $\Delta f_{i}=0\;MHz$, and the one-dimensional range image of the tested radar false alarm signal is shown in Figure 2. According to Equations (10) and (14), the waveform of false alarm signal is broadened due to the existence of secondary phase.

**Figure 3:**
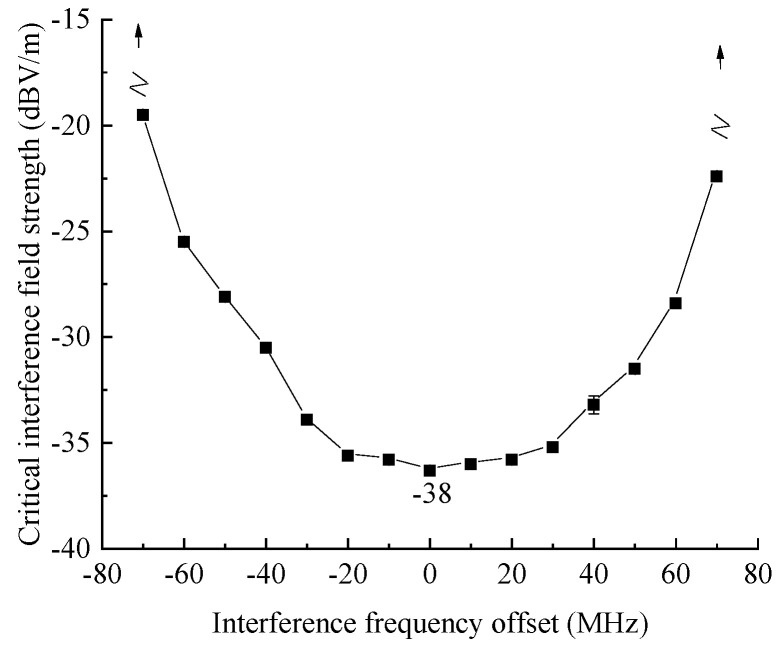
Results of false alarm sensitive threshold of test equipment.

Firstly, combined with the definition of false alarm signal in the early stage, it can be known that the absolute level of false alarm signal is higher than $u=20\mathrm{lg}\left[5\times 2\times 10^{3}/\left(2\times 2^{12}\right)\right]\approx 2\;dBmV$, which is regarded as the effective false alarm level, the electromagnetic sensitivity threshold test has been conducted on the test equipment in the previous period, and the result is shown in Figure 3, which shows that the equipment sensitivity bandwidth is about plus or minus 75 MHz [8]. Therefore, exploring the position of the false alarm signal of the tested radar, set the interference frequency offsets as Δ*f*_0_ = 0 MHz, Δ*f*_1_ = −60 MHz, Δ*f*_2_ = 60 MHz, Δ*f*_3_ = −75 MHz, Δ*f*_4_ = 75 MHz corresponding to the position of the false alarm signal as *R*_0_, *R*_1_, *R*_2_, *R*_3_, *R*_4_, respectively, as shown in Table 2.

It can be seen from Table 2 that when the Δ*f*_0_ = 0 MHz, the position *R*_0_ of the false alarm signal changes irregularly. Under different interference frequency offsets, the position *R* of false alarm signal shows the same characteristics. Because the selected frequency points are representative, it is inferred that the position of a single-frequency electromagnetic false alarm signal in the radar band of the tested radar will show similar effect rules. Combined with Equation (14), the initial phase of the local oscillation signal of the tested radar changes irregularly with the detection period *k*.

### 4.3. Characteristic Test Results and Analysis of Dual-Frequency Non-Intermodulation False Alarm Interference

Firstly, the waveform characteristics of the false alarm target in the test radar under dual-frequency electromagnetic interference are observed. Set the interference frequency bias Δ*f_j_*_1_ = −0 MHz and Δ*f_j_*_2_ = 40 MHz of the interference signal, and the one-dimensional distance of the target after detection is obtained as shown in Figure 3.

**Figure 4:**
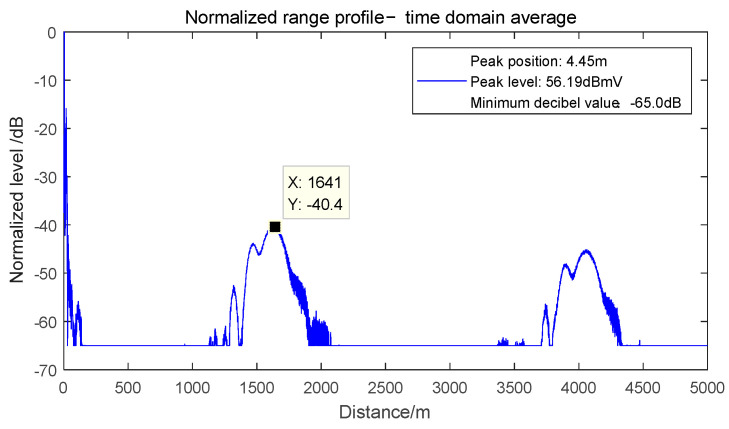
Position of false alarm signal under Δ*f_j_*_1_ = 0 MHz and Δ*f_j_*_2_ = 40 MHz electromagnetic inter-ference.

It can be seen from Figure 4 that two false alarm targets appear when the tested radar is interfered by the in-band dual frequency, and the waveform of the false alarm signal is broadened, which is consistent with the theoretical analysis of Equation (21). Next, analysis the influence of dual frequency electromagnetic interference on false alarm signal position. To distinguish the two interference signals, the field strength difference between the two interference components is set to be more than 6 dB.

The two interfering frequency points are selected with attention to avoid the point of in-band intermodulation. From the previous analysis, it is clear that the unblurred distance corresponding to the tested radar transmitting sub-period is 7500 m, and the tested radar terminal display interface range is up to 5000 m. If the false alarm target position is in 5000~7500, the display interface cannot be observed. The purpose of the experiment is to verify the theoretical analysis results. Therefore, the actual occurrence positions *R*_1_′ and *R*_2_′ of the two false alarm signals can be recorded when the two false alarm signals are displayed as much as possible through multiple detections, and the results are shown in Table 3.

It can be seen from Table 3 that dual-frequency electromagnetic interference forms two false alarm signals, and the position of any false alarm signal appears randomly, which is consistent with the analysis of Equations (21) and (25). When two false alarm targets appear, they may not appear in the terminal one-dimensional range image display window. However, when two appear at the same time, in the above ten measurements, the average value is −2898 m when the position appears near −2925 m. By substituting the frequency offset of the two interference signals into Equation (26), the calculated distance difference is −2925 or 4575. The experimental results are in agreement with the theoretical analysis.

Similarly, the interference component 2 frequency bias is increased, and the results are shown in Table 4.

From Table 4, it can be concluded that when the interference frequency offset increases, in the above ten measurements, the average value is −2935 m when the position appears near −2925 m, and the experimental data is consistent with the theoretical calculation. The test data is consistent with the theoretical calculation results. It shows that the distance value between the dual-frequency non-intermodulation false alarm interference signals of the tested radar under other interference frequency points shows similar characteristics. The test results are consistent with the theoretical analysis.

### 4.4. Test Results and Analysis of Radar Second-Order Intermodulation False Alarm Interference

**Figure 5:**
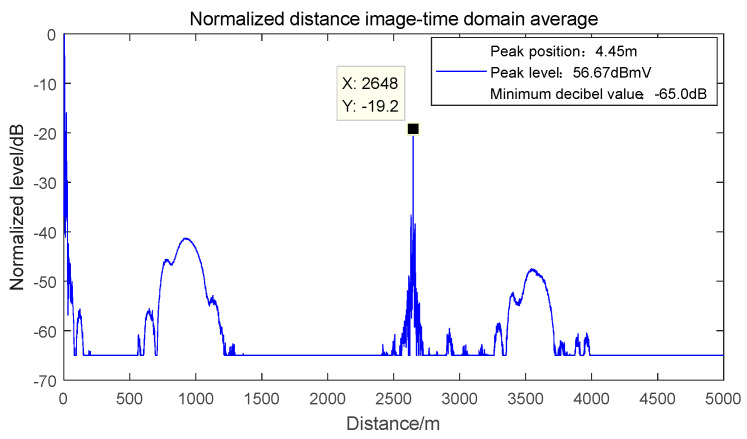
One-dimensional range profile of second-order intermodulation false alarm signal.

The following continue to explore the second-order intermodulation false alarm target location law through the test, as shown in Figure 5 “spike” type second-order intermodulation false alarm signal. Continuous multiple detection records the actual location of the intermodulation signal. The results are shown in Table 5.

According to the Formula (30), calculate the actual appearance position *R_c_*′ of the false alarm signal without considering distance collapse. According to the Formula (21) calculate the false alarm signal position *R_t_*′ after distance folding and compare *R_jm_*_2_, *R_c_*′, and *R_t_*′. The results are shown in Table 6.

As can be seen from Table 6, when Δ*f_j_*_2_ is 1.65 kHz, 3.60 kHz, and 6.60 kHz, the position of second-order intermodulation false alarm signal increases gradually, which is consistent with R theory calculated by Equation (30). As the value of interference frequency offset Δ*f_j_*_2_ continues to increase, the target position of second-order intermodulation false alarm seems to decrease gradually and then increase. According to the research content on distance “reentry” in literature [16], when the false alarm signal exceeds the sub-period non-fuzzy distance value of 7500 m, the ‘retrace’ occurs. After the original distance value is calculated according to the theoretical equation, the final value is the actual position of the second-order intermodulation false alarm intermodulation according to the unambiguous distance corresponding to the sub-period. Comparing the measured *R_av_* with the *R_t_*′ theory, it can be seen that the experimental results are consistent with the theoretical analysis. The test data are stable, with small relative error and good repeatability, the test equipment does not appear dead, and restarted during the test.

## 5. Conclusions

This paper takes a stepped frequency radar as the research object. Firstly, use the stretch processing method to decouple the radar received signal in time and frequency, distinguish the target echo signal from false alarm interference signal, and analyze the target characteristics of false alarm interference theoretically. Secondly, from the perspective of receiver mixing, the target echo signal and false alarm interference signal are distinguished, and the imaging characteristics of single-frequency, dual-frequency non-intermodulation, and second-order intermodulation false alarm signals are analyzed. Finally, the electromagnetic interference test of the tested radar is carried out by the electromagnetic injection method. The following conclusions are drawn:

1. The electromagnetic sensitive bandwidth of the false alarm signal is about ±75 MHz, which is smaller than its working bandwidth.
2. The position of the single-frequency electromagnetic false alarm interference signal of the tested radar is affected by the local oscillator phase, and the position shows random performance.
3. Without considering the intermodulation, the dual-frequency electromagnetic interference makes the test radar generate two false alarm signals with wide waveform. The dual frequency non-intermodulation false alarm signal distance difference is related to the interference frequency difference. Under the condition of tight constraints, the frequency offset of dual-frequency interference can be substituted into the test radar according to Formula (26) to obtain the distance difference between the two false alarm targets of −2925 m and 4575 m, and the test results are consistent with the theory.
4. The second-order intermodulation false alarm signal is ‘spike’ shaped; its position is related to the frequency offset of dual-frequency interference, the relative error is less than 0.1, and the test data is stable.

## 6. Discussion

Firstly, through the study of the electromagnetic interference false alarm signal, it is found that the radar is affected by external electromagnetic interference. When the frequency offset of the interference signal is close to the frequency of the working signal, the interference signal and the useful signal enter the receiver and participate in the process of mixing, amplification, and filtering. Next, the false alarm signal is generated by signal processing, which affects the judgment of the received signal. When the deviation range between dual-frequency electromagnetic interference is small to a certain extent, it will produce second-order intermodulation false alarm interference, which also affects the judgment of target echo signal. Although many scholars have proposed corresponding anti-interference algorithms for radar external electromagnetic interference, due to the characteristics of radar itself, the signal frequency after mixing may not be fixed, which makes the filter unable to effectively filter out the interference signal. This paper analyzes the law of false alarm interference position of the tested radar, which provides strong support for the modeling of multi-frequency electromagnetic false alarm interference in the next step.

Secondly, when the interference frequency is close to the operating frequency, in addition to generating second-order low-frequency intermodulation interference, the mixing frequency will also generate third-order intermodulation false alarm signals. The third-order intermodulation false alarm signal target characteristics and position change law are the next planned focuses.

In practical applications, when the external interference increases to a certain degree, the nonlinearity of the device will cause the radar to generate false alarm signals while suppressing the target echo level, and the generation of higher-order intermodulation false alarm signals will affect the capture of real target signals, so it is necessary to conduct a detailed study to explain the intermodulation false alarm signals and establish a multifrequency electromagnetic false alarm interference signal model on this basis to explore the radar equipment. This is also the focus of the next work.

## Figures and Tables

**Table 1:** Imaging features of false alarm signal.

False Alarm Signal Characteristics	Single-Frequency	Dual-Frequency	
		Non-Intermodulation	Second-Order Intermodulation
Waveform	Spike type	Waveform broadening	Spike type
Position	*R_j_*_1_′	Δ*R* = *R_j_*_1_′ − *R_j_*_2_′	*R_jm_*_2_

**Table 2:** Position of false alarm signal under single frequency electromagnetic interference.

*E_j_*/(dBV/m)	*R*_0_/m	*R*_1_/m	*R*_2_/m	*R*_3_/m	*R*_4_/m
−19	2676	4404	3913	1594	1829
−16	1428	2510	3463	2226	1371
−12	2906	2455	2003	4103	2081
−9	626	3988	3645	4573	1432
−6	1085	1823	1738	546.7	4544
−3	3149	2287	4198	2533	4376
0	503	999.1	3187	3505	1258
3	1368	932	4924	1654	2137
6	2550	263	4908	3587	710.9
9	2708	1834	1131	1767	1924
12	1172	437	4075	1822	431.9

**Table 3:** The position of false alarm signal under Δ*f_j_*_1_ = 0.02 kHz and Δ*f_j_*_2_ = 3.92 kHz.

Serial Number	*R*_1_′/m	*R*_2_′/m	Δ*R*/m
1	4771	1876	−2895
2	4963	2083	−2880
3	3694	775	−2919
4	4182	1277	−2905
5	4318	1423	−2895
6	328	4923	4595
7	3017	128	−2889
8	3535	640	−2895
9	3179	278	−2901
10	4081	1176	−2905

**Table 4:** The position of false alarm signal under Δ*f_j_*_1_ = 0.02 kHz and Δ*f_j_*_2_ = 13.92 kHz.

Serial Number	*R*_1_′/m	*R*_2_′/m	Δ*R*/m
1	3647	701	−2946
2	4761	1815	−2946
3	3668	746	−2922
4	4818	1888	−2930
5	3886	943	−2943
6	230	4791	4561
7	4263	1320	−2943
8	4795	1877	−2918
9	4293	1378	−2915
10	3336	381	−2955

**Table 5:** Second-order intermodulation signal position under different frequency offset.

Δ*f_j_*_2_/kHz	*R_jm_*_2_/m					*R_av_*/m
1.65	1270	1270	1270	1271	1269	1270
3.60	2718	2722	2721	2719	2723	2721
6.60	4936	4932	4928	4927	4920	4929
14.51	4113	4115	4118	4120	4120	4117
18.51	1150	1152	1153	1155	1153	1153
20.57	350	349	349	349	348	349
23.50	2596	2596	2593	2594	2592	2594
26.38	4839	4835	4834	4833	4833	4835
58.38	1210	1210	1211	1211	1211	1211
84.38	3280	3278	3278	3278	3277	3278

**Table 6:** Measured and theoretical data under different frequency offset.

Δ*f_j_*_2_/kHz	*R_av_*/m	*R_c_*′/m	*R_t_*′/m
1.65	1270	1238	1238
3.60	2721	2700	2700
6.60	4929	4950	4950
14.51	4117	10,883	4118
18.51	1153	13,883	1118
20.57	349	15,360	360
23.50	2594	17,625	2625
26.38	4835	19,785	4785
58.38	1211	43,785	1215
84.38	3278	63,285	3285

## Data Availability

The authors confirm that the data supporting the findings of this study are available within the article.
